# General Image Fusion for an Arbitrary Number of Inputs Using Convolutional Neural Networks

**DOI:** 10.3390/s22072457

**Published:** 2022-03-23

**Authors:** Yifan Xiao, Zhixin Guo, Peter Veelaert, Wilfried Philips

**Affiliations:** Department of Telecommunications and Information Processing, IPI-IMEC, Ghent University, 9000 Ghent, Belgium; zhixin.guo@ugent.be (Z.G.); peter.veelaert@ugent.be (P.V.); wilfried.philips@ugent.be (W.P.)

**Keywords:** image fusion, multiple inputs, permutation-invariant network, continual learning

## Abstract

In this paper, we propose a unified and flexible framework for general image fusion tasks, including multi-exposure image fusion, multi-focus image fusion, infrared/visible image fusion, and multi-modality medical image fusion. Unlike other deep learning-based image fusion methods applied to a fixed number of input sources (normally two inputs), the proposed framework can simultaneously handle an arbitrary number of inputs. Specifically, we use the symmetrical function (e.g., Max-pooling) to extract the most significant features from all the input images, which are then fused with the respective features from each input source. This symmetry function enables permutation-invariance of the network, which means the network can successfully extract and fuse the saliency features of each image without needing to remember the input order of the inputs. The property of permutation-invariance also brings convenience for the network during inference with unfixed inputs. To handle multiple image fusion tasks with one unified framework, we adopt continual learning based on Elastic Weight Consolidation (EWC) for different fusion tasks. Subjective and objective experiments on several public datasets demonstrate that the proposed method outperforms state-of-the-art methods on multiple image fusion tasks.

## 1. Introduction

Image fusion creates a composite image by merging multiple source images from different modalities or different camera settings of the same scene. It contains a series of fusion tasks, such as multi-focus image fusion, multi-exposure image fusion, multi-modality medical image fusion, and infrared/visible image fusion. Image fusion helps to obtain better visual performances for the fused image. For example, the multi-exposure image fusion produces a high dynamic range (HDR) image by combining a series of low dynamic range (LDR) images, breaking the limitation of the camera exposure range. Multi-focus image fusion extends the field-of-view for optical cameras by fusing defocused images to an all-in-focus image with clear content. Besides, image fusion also helps to obtain richer information from different sensors, which benefits some particular applications. For instance, multi-modal medical image fusion integrates different tissues information from the Magnetic Resonance Imaging (MRI) images and Positron Emission Tomography (PET) images to assist the clinical diagnosis of doctors. In addition, fusing visible images with infrared images in night vision can acquire enhanced and more detailed scene information, which benefits the detection and tracking.

**Figure 1:**
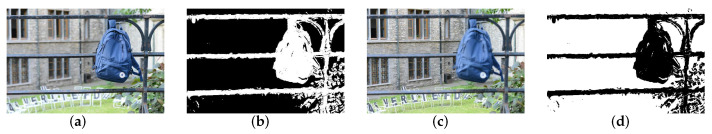
The source defocused images (**a**,**c**) and the corresponding activity level measurements (**b**,**d**).

Existing literature on image fusion can be roughly categorized into traditional framework-based image fusion methods and deep learning-based image fusion methods [1]. Most traditional framework-based methods concentrate on two techniques for image fusion, namely image activity level measurement and fusion rule design. The activity level represent the important degree of each pixel in the source images, such as the saliency of pixels in infrared/RGB images [2] or the focused degree of pixels in defocused images [3], etc., as shown in Figure 1. Usually, the measurement accuracy of the activity level determines the quality of the fusion image. Various powerful signal processing tools have been used to measure the activity level, such as the multi-scale decomposition and sparse representation. However, we place higher demands on the image representation ability of the algorithm to get more accurate activity level measurement. Deep learning (DL) has shown potent feature extraction and representation capability than classical algorithms. Therefore, the DL-based methods are now widely explored in image fusion tasks [4]. The DL-based methods construct specialized neural networks for each fusion application. They often generate high-quality fused images, such as Densefuse [5] for infrared/visible image fusion, DRPL [6] for multi-focus image fusion, and DeepFuse [7] for multi-exposure image fusion. Despite better fusion performance, a challenge exists in DL-based fusion methods: existing DL-based methods only support a fixed number of inputs (typically two inputs). Due to their structural immutability, neural networks require the same number of inputs during inference as in the training process. When handling more than two inputs, most DL-based methods fuse the images recursively, which losses the unified consideration of all input images and is time-consuming [8]. However, many fusion tasks, such as multi-focus image fusion and multi-exposure image fusion, usually contain an unfixed number of inputs. To solve the problems mentioned above, we present a novel general image fusion method capable of different fusion tasks with an arbitrary number of inputs. We name it IFANI, i.e., general Image Fusion method for an Arbitrary Number of Inputs. Several advantages of the proposed IFANI are highlighted in the following:

- **Unified**. Different from the specialized frameworks designed for one specific fusion task, this is a generic fusion framework that can handle a diversity of fusion tasks by a single model trained with a continual learning strategy.
- **Flexibility**. The flexibility of the framework is embodied in two aspects: (1) It can handle an arbitrary number of inputs, which differs from DL-based methods designed for fixed inputs. (2) It is a permutation-invariant fusion network that can effectively fuse the informative details without remembering the order of the input images. To achieve that, we adopt the symmetry function max-pooling, which can fuse a variable number of inputs into one output feature map without considering the order of inputs and enable permutation-invariant of the network. The max-pooling is repeatedly used in different stages of the network to aggregate salient information among all the inputs. The fused image is reconstructed through convolutional layers from the output pooled feature map. Additionally, skip connections aid the reconstruction of details at different scales.
- **Trainable**. Due to the lack of large multi-focus images training datasets, we propose a simulation method for arbitrary focus-complementary images to generate adequate training data for the multi-focus image fusion task.
- **Quality**. We subjectively and objectively conduct experiments on four tasks, including medical image fusion, infrared/visible image fusion, multi-exposure image fusion, and multi-focus image fusion. The experimental results show that our fused images have comparable or better quality than the state-of-the-art while being more widely applicable by not requiring retraining.

The remainder of this paper is organized as follows. In Section 2, we give a survey about image fusion methods and introduce the permutation-invariance of the network. Section 3 describes the proposed image fusion method in detail. Section 4 gives the experimental results and the ablation study. Section 5 concludes this paper.

## 2. Related Work

Existing fusion methods can be generally categorized into two types: traditional image fusion methods and deep learning-based image fusion methods. We will first review these two types of fusion methods, then introduce basic knowledge about the permutation-invariant networks related to our work.

### 2.1. Traditional Image Fusion Methods

In traditional image fusion methods, the key factors include how to measure the pixel activity level of each source image and to design a suitable fusion strategy to obtain the fused image. According to the way to measure the pixel activity level, the traditional image fusion methods can be categorized into transform domain methods, spatial domain methods, and hybrid methods combining these two. The transform domain methods measure the image activity level by the coefficients in a transformational domain such as pyramid transform [9], wavelet transform [10], edge-preserving decompositions [11], and Shearlet transform [12]. The fused image is reconstructed by the inverse transform based on the fused coefficients. Also, based on the transform domain, the sparse representation method can efficiently represent the saliency information of the original images with the sparse coefficients but at the price of higher computational complexity [13,14]. Different from transform domain methods, spatial domain methods directly compute the image activities without transferring to other domains [15]. The activity level of images can be computed at the pixel [16], block [17] or region [18] level. These methods are efficient but may lead to block effects or halo edge for the reconstructed image. The hybrid methods integrate the strengths of different transform or spatial domain methods. For instance, Liu et al. [19] proposed a hybrid image fusion method by combining multi-scale transform and the sparse representation. Experiments show that the combined method usually performs better than using the individual technique. Even so, manual feature extraction methods are still insufficient to represent the subtle details of the images, leading to inaccurate activity level measurement.

### 2.2. Deep Learning-Based Image Fusion Methods

Convolutional neural networks (CNNs) show high performances on various fusion tasks [4]. Intuitively, a CNN can be used as the feature extractor to obtain the activity level measurement results of the source images [5,20]. But an end-to-end deep learning framework integrating both activity level measurement and fusion rule has become the tendency for image fusion recently. Various end-to-end models have been proposed to deal with multi-modal image fusion, such as infrared/visible image fusion. For instance, FusionGAN [21] takes advantage of the generative adversarial network (GAN) to obtain a fused image with infrared intensity and visible details. Xu et al. [22] extend FusionGAN with two discriminators to preserve more meaningful information of the source images, while Li et al. [23] enhance the fusion ability of the network by designing the coupled GAN. GAN-based method [24] is also applied to the fusion of multi-focus images, while more DL-based multi-focus image fusion methods adopt a classification model or a regression model for their frameworks [3]. In the classification strategy, the focused pixels and defocus pixels are defined as two different categories. The CNNs discriminate between these two categories and fuse the pixels belonging to the focused category to obtain the all-in-focus image. Tang et al. [25] and Liu et al. [26] train the network with simulated blur/clear image patches and output the focus maps of corresponding source images. Amin-Naji [27] and Ma et al. [28] improve the classification accuracy by ensemble learning and consistency verification techniques and generate the all-in-focus image with refined focus maps by post-processing. Li et al. [6] directly convert the whole image into a binary mask without any patch operation and obtain the fused image by multiplying the sources and the corresponding binary masks. This method integrates focus map generation and all-in-focus image fusion together and avoids complex post-processing. Such an end-to-end regression model is also applied in [29], where the U-net improves the ability of feature extraction and fuses the all-in-focus image with a clear boundary. As for the multi-exposure images, DeepFuse [7] is the first DL-based multi-exposure image fusion method. It extracts features from the low-exposure image and the high-exposure image with two branches of the encoder. These features are then fused to reconstruct the HDR image by a decoder. In this framework, an ingenious non-reference metric designed for the multi-exposure images fusion, MEF-SSIM [30], is applied as the loss function to guide the convergence of the model. Ma et al. [31] speed up the fusion process by a down-sampling/up-sampling process. They first feed a low-resolution version of the input sources to a fully CNN for weight map prediction, then jointly up-sample the weight maps by a guided filter and obtain the fused image by a weighted fusion. Although the above methods achieve good performance, they are specially designed for a single fusion task and usually can not apply for another fusion task.

Some researchers have proposed to use one framework to handle different fusion problems. IFCNN [32] trains end-to-end neural networks on a large-scale RGB-D dataset that provides ground truth fused images. It can conduct inferences on various fusion tasks, such as medical, infrared/visible, multi-focus, and multi-exposure image fusions. However, IFCNN only trains multi-focus images synthesized by the RGB-D dataset, lacking knowledge about other fusion tasks. FusionDN [33] and U2Fusion [8] conduct continual learning on several fusion tasks. They adopt the elastic weight consolidation [34] to preserve information from the previous task when training on the new task, which also inspires our work. However, they can only handle two inputs. When there are three or more inputs, they need to recursively fuse a new input with the previously fused image, ignoring the global vision for all images. This recursive fusion extracts the feature of the previous fused image repetitively, which is time-consuming and does not suit a larger number of inputs. In reality, fusion tasks such as multi-exposure and multi-focus image fusion always contain more than two images. To handle the above challenges, we propose an end-to-end CNN framework that can fuse an arbitrary number of inputs at once for different fusion tasks. Our method takes the permutation-invariance of the network. It does not need to remember the specific order of different inputs but efficiently fuses distinct and important information of different images. Such permutation-invariance makes it possible for our network trained with *M* inputs to be applied for *N* inputs (M,N≥2andM≠N). Before introducing our proposed method, we first introduce the concept of permutation-invariance of networks.

### 2.3. Permutation-Invariant Networks

For most neural networks, the order of inputs is a part of the information, which means switching the order of inputs usually changes the outputs. For example, the recursive neural network understands the sentence meaning through the order of words, and switching the order of words usually changes the meaning of the sentence. However, there is a type of problem concerning unordered sets, for which changing the order of the items should not change the result. One typical example is the point clouds. Point clouds have the property of unordered that each point position has its set of Cartesian coordinates. Any global properties computed from cloud points should not depend on their input order but the coordinates data. Without specialized design, a CNN will attribute some meaning to the input order, which is unfavorable. Researchers proposed various methods to enforce a network to be permutation-invariant for unordered inputs to handle this problem.

Zaheer et al. [35] theoretically define the characteristics of permutation-invariant functions and define a family of functions with permutation-invariant properties, termed as symmetry functions. They also prove through theory and experiment that max-pooling and sum-decomposition operations effectively solve the permutation-invariance problem of the neural networks. Accordingly, Qi et al. [36] construct a permutation-invariant network PointNet using max-pooling to handle the tasks of object recognition and scene semantic word parsing. Herzig et al. [37] add sum-decomposition to the hidden layer to make the network meet the permutation-invariance and successfully achieve new state-of-the-art results on the Visual Genome scene-graph labeling benchmark. Aittala et al. [38] design a permutation-invariant network by symmetrical max-pooling to denoise a sequence of Burst images.

**Figure 2:**
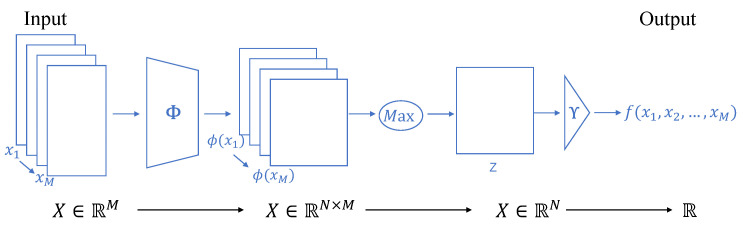
Model structure proposed in related work for representing permutation-invariant functions. The Max operation enforces permutation-invariance for the model. ϕ and γ can be implemented by multilayer perception, and *Z* is latent space.

As max-pooling is a popular choice for symmetric functions, below, we will introduce how max-pooling is used in CNN to enable permutation-invariance. The pipeline is illustrated in Figure 2. Mathematically speaking, given an unordered set $X\in R^{M}$ with items of $x_{1},x_{2},\ldots ,x_{M}$, we say that a function *f* defined on the set *M* is *max-decomposable via* *Z* if there are functions ϕ:R→Z and γ:Z→R such that:

(1) $$f\left(X\right)=\gamma \left(\max_{i}\phi (x_{i})\right)\;,$$

where we refer to *Z* as the *latent space* and the max is taken over each dimension independently in the latent space. Since max is permutation-invariant, a max-decomposition is also permutation-invariant [39]. In terms of neural networks, ϕ and γ can be realized by multilayer perceptrons. This framework firstly extracts features of every element in an unordered input set individually. Then, these features are pooled by evaluating the maximum value of each feature across the members. After the pooling operation, the original representations of the input individual are discarded, while the pooled feature illustrating the most vital information of the inputs are maintained and are processed by subsequent networks. Finally, the neural network outputs the desired image or probability distribution by the last multilayer perceptrons. From the above, we can see that max-pooling plays the role of feature fusion. Because of its permutation-invariance, the network’s output has nothing to do with the order of the inputs. In our fusion task, the network should output high-quality fused images regardless of the number or order of the input images. Thus, we adopt the symmetry function max-pooling to achieve the permutation-invariance of our network. Compared with sum-pooling, max-pooling is efficient in extracting distinguishing features of each input; however, it is sensitive to outliers. Considering this problem, we take a concatenate strategy to mitigate the effects of outliers of the network, which will be introduced in the succeeding section. Experiments also prove the efficiency of max-pooling than sum-pooling in our framework for the image fusion (Section 4.5.1).

## 3. Proposed Method

Our method aims at fusing an arbitrary number of images for different fusion tasks by a unified model, breaking the limitation of existing DL-based fusion methods designed for a fixed number of images. We introduce the permutation-invariance to our neural network, which enables the network to extract and fuse informative features without deliberately remembering the order of input images. This section presents the network architecture, the training data generation method, the continual learning method, and the implementation details.

### 3.1. Network Architecture

**Figure 3:**
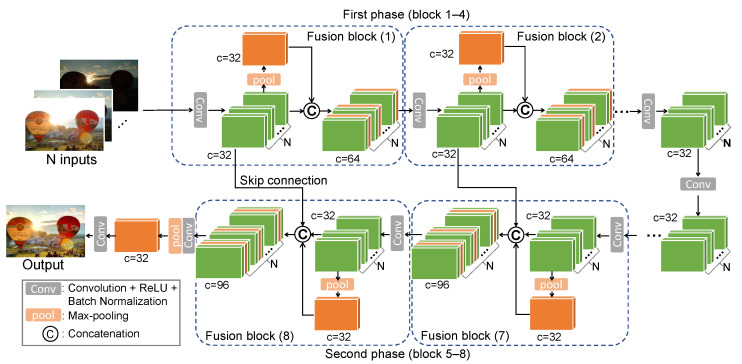
Framework of the proposed IFANI for multiple image fusion tasks. Here we take multi-exposure image fusion, for example. The inputs contain *N* frames of images, which are processed by a *Conv* process to get *N* corresponding feature maps with *c* channels in each. The network has eight fusion blocks, each of which aggregates the most informative information among *N* feature maps by max-pooling and fuses this aggregated feature map with the *N* respective feature maps. The *Conv* module in each fusion block is composed of a convolutional layer, a ReLU, and a batch normalization layer, where the size of all convolutional kernels is 3×3.

The overall architecture of IFANI is presented in Figure 3. Our IFANI adopts a symmetrical structure, containing 4 fusion blocks in the first and second phases, respectively. Besides, a bottleneck connects the first and second phases and the tail part outputs the final fused image. Suppose there are *N* input images (N≥2). In the first fusion block of the first phase, the network extracts the features of each image by a *Conv* process composed of a convolutional layer, a Rectified Linear Unit (ReLU), and a batch normalization layer. The feature extraction is realized by a *batch-mechanism* operation, which can be seen as one *Conv* module sequentially processing *N* inputs, or *N* identical *Conv* modules processing *N* inputs in parallel. No matter which implementation is used, it essentially learn one filter kernel instead of *N* different kernels. This *batch-mechanism* operation is applied for all subsequent *Conv* modules. Then, the *N* feature maps are pooled by max-pooling to obtain the most prominent features among them. We concatenate the original *N* feature maps with this pooled feature so that every feature map gains information from other feature maps, which plays a role in information fusion. The concatenated feature maps are fed to the next fusion block, and we repeat the same operation until the end of the first phase (the 4-th fusion block). The second phase is composed of 4 similar fusion blocks. The difference is that feature maps in the first phase will be reused through skip connections for feature map concatenation, preventing the network from losing low-level details of the input images. A bottleneck module composed with two *Conv* plus one more convolutional layer connects the firs and the second phases. After the 8-th fusion block, we feed the last group of concatenated feature maps into a *Conv* module to reduce their channels from 96 to 32. Then we apply the max-pooling to obtain the last fused feature map, which reconstructs the fused image through the last *Conv* module. The filter size and trainable parameters of each layer are illustrated in Table 1.

The ideal method of image fusion is processing the source image by assigning them preferences according to their characteristics. However, it requires the same number and order of images during training and inference, making it inconvenient when the inputs are unfixed. Our IFANI acquires the property of permutation-invariance by the max-pooling, which means the order of inputs does not affect the fusion performance so that network does not need to remember the order of different inputs. In addition, the *batch-mechanism* operation helps the network process a variable number of images. Therefore, even trained with *M* images, the network can be also applied for *N* images fusion. Moreover, the skip connection between the first and second phases helps maintain image details in the fused images. Although without remembering the order of input images, experiments in Section 4 prove that our method outperforms others.

### 3.2. Continual Learning on Multiple Image Fusion Tasks

**Figure 4:**
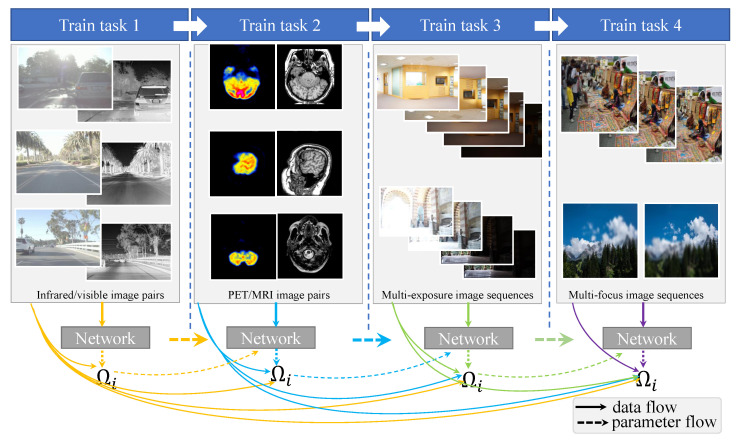
Continual learning for four different fusion tasks. The solid lines below represent the data flow. The thick solid lines represent the data used to train the network. The thin solid lines represent a small subset of data used to calculate the importance parameter $\Omega_{i}$. The dotted lines indicate that the network parameters are passed to the training of the next task.

We implement our IFANI for four image fusion tasks, including multi-modal medical image fusion (task 1), infrared/visible image fusion (task 2), multi-exposure image fusion (task 3) and multi-exposure image fusion (task 4). The intuitive idea of handling multiple image fusion tasks by one model is to mix all training data into a large training set and train the network on this big dataset. However, these tasks contain four types of source image sets, which are diverse and have different data distributions. If we change the type of input image at every iteration during training, the parameters of the network will change drastically, and the training will be difficult to converge. To make the network learn from every task and converge quickly, our IFANI is sequentially trained from task 1 to task 4 with a continual learning mechanism based on the Elastic Weight Consolidation [40], which will be introduced in Section 3.2.2. The training procedure is shown in Figure 4.

For four fusion tasks, the loss function comprises the image quality assessment (IQA) function and the loss function used for continuous learning, in which the IQA metric includes reference-based measurement and non-reference measurement, as formulated below:

(2) $$L_{n}=\lambda_{1}L_{\mathrm{ref}}+\lambda_{2}L_{\mathrm{nref}}+\lambda_{3}L_{\mathrm{ewc}}\;.$$

We mark the *n*-th task as $T_{n}$, then $L_{n}$ is the loss function used in $T_{n}$. $L_{\mathrm{ref}}$ and $L_{\mathrm{nref}}$ represent the reference-based IQA and non-reference-based IQA, respectively. $L_{\mathrm{ewc}}$ is the loss function based on Elastic Weight Consolidation [40], which overcomes catastrophic forgetting of the previously trained tasks when training on a new task. The weight λ for each loss function balances the penalty between the IQA loss and the loss of continual learning.

#### 3.2.1. Loss Function of Image Quality Assessment

We adopt a widely used loss function, mean absolute error, as the reference-based IQA to evaluate the quality of the fused image:

(3) $$L_{\mathrm{MAE}}\left(Y,\hat{Y}\right)=\frac{1}{whc}\sum_{x,y,k}\left|Y_{x,y,k}-{\hat{Y}}_{x,y,k}\right|\;,$$

where Y and $\hat{Y}$ are the fused image and the reference ground truth. *w*, *h*, and *c* represent the image’s width, height, and the number of channels. *x*, *y*, and *k* are pixel coordinates.

For fusion tasks such as medical image fusion and infrared/visible image fusion, their training data lack the reference ground truth for supervision. Therefore, direct use of the similarity index measure (SSIM) is impossible, which requires a single perfect quality reference image. A non-reference IQA function is commonly used for quality measurement of the fused image. To guide the network to fuse meaningful structures and details from every source image, we adopt MEF-SSIM [30] as the non-reference IQA loss function. MEF-SSIM is proposed based on the principle of the structural similarity (SSIM) [41] approach as well as a novel measurement of patch structural consistency between multiple source images and the the fused image, which is firstly used to assess the image quality of the multi-exposure image fusion. Different from the SSIM, MEF-SSIM does not need the ground truth image as reference. If a fused image integrates more structured information from the source images, it has a higher MEF-SSIM score. MEF-SSIM gets the assessment results that match the subjective judgements well and is therefore widely used on unsupervised learning of multi-exposure fusion networks [7,31]. MEF-SSIM is also used in the objective quality comparison of other fusion tasks, such as infrared/visible fusion [5], and shows consistency with the subjective measurements. The loss function based on MEF-SSIM is defined as:

(4) $$L_{\text{MEF-SSIM}}\left(X,Y\right)=1-\text{MEF-SSIM}\left(X,Y\right)\;,$$

where X represents source images and Y is the fused image.

#### 3.2.2. Loss Function of Elastic Weight Consolidation

To handle different fusion tasks with one unified model, we take advantage of the redundancy of network parameters and integrate characteristics of different tasks by continual learning. When sequentially training multiple tasks, we expect the learned knowledge from the previous task $T_{n-1}$ can boost the learning of current task $T_{n}$. Also, we hope the model will not lose the knowledge from task $T_{n-1}$ after the training of task $T_{n}$. Continuous learning by Elastic Weight Consolidation [40] provides a solution that accumulates knowledge over tasks and overcomes catastrophic forgetting about previous knowledge by the regularization-based method on the parameters of the network. Specifically, the model parameters trained with the previous task $T_{n-1}$ and current task $T_{n}$ are marked as $\theta^{*}$ and θ, respectively. When a new task $T_{n}$ to be learned, we have in addition to the new task loss $L_{n}\left(\theta \right)$, a regularizer $L_{\mathrm{ewc}}$ that penalizes changes to parameters that are deemed important for the previous task

(5) $$\begin{array}{c} L(\theta )=L_{n}\left(\theta \right)+L_{\mathrm{ewc}}\\ \;\;\;=L_{n}\left(\theta \right)+\eta \sum_{i}\Omega_{i}{\left(\theta_{i}-\theta_{i}^{*}\right)}^{2}\;, \end{array}$$

with a penalty factor η that sets how important the old task is compared to the new one. $\theta_{i}$ represents the *i*-th parameter in the network. $\Omega_{i}$ is the importance degree of each old parameter $\theta_{i}^{*}$, which guides the network to change the parameters that are not important to the previous task (low $\Omega_{i}$) but limit the change of important parameters (high $\Omega_{i}$). The importance matrix Ω can be computed based on any available data considered most representative for test conditions. Here we follow the importance measurement approach in [40], which is based on Fisher information matrix, and the $\Omega_{i}$ is estimated by:

(6) $$\Omega_{i}=\frac{1}{N}\sum_{d\in D^{*}}\frac{\partial L{\left(d,\theta^{*}\right)}^{2}}{\partial \theta^{*}}\;,$$

where *d* represents the elements in dataset $D^{*}$ of the previous task and the total elements is *N*. The EWC loss in the current task should be

(7) $$L_{\mathrm{ewc}}=\eta \frac{1}{N}\sum_{i}\sum_{d\in D^{*}}\frac{\partial L{\left(d,\theta_{i}^{*}\right)}^{2}}{\partial \theta_{i}^{*}}\left(\theta_{i}-\theta_{i}^{*}\right)\;.$$

Note that $\Omega_{i}$ is calculated once the previous task has been trained, and the training of the current task does not require the data of the old task. Besides, for several tasks, the importance degree $\Omega_{i}$ will accumulate along with the tasks. As shown in Figure 4, a small subset dataset in the current dataset is used to calculate the importance degree for the next task. Take task 3 for example, $\Omega_{i}$ in task 3 are calculated by Formula (6), where $D^{*}$ are subsets data from task 1 and 2. $\theta^{*}$ are parameters in network trained by task 2.

Overall, if the fusion task has ground truth for training (such as multi-exposure and multi-focus image fusion tasks in our papers), its loss function can be formulated as:

(8) $$L=\lambda_{1}L_{\mathrm{MAE}}+\lambda_{2}L_{\text{MEF-SSIM}}+\lambda_{3}L_{\mathrm{ewc}}\;,$$

where we use both reference and non-reference quality metrics to measure the fused image. In the training process the parameters $\lambda_{1}$ and $\lambda_{2}$ are set to 0.5 so that the contribution of the two losses would be roughly balanced. Since the value of $L_{\mathrm{ewc}}$ is much smaller than $L_{\mathrm{MAE}}$ and $L_{\text{MEF-SSIM}}$, the order of magnitude of $\lambda_{3}$ is particularly important, which is defied at $10^{4}$ after the investigation. We also notice that the results are not significantly sensitive for $\lambda_{3}$ varying from $1\times 10^{4}$ to $3\times 10^{4}$ but will drop down when $\lambda_{3}$ is bigger than $3\times 10^{4}$. Therefore, we finally set the weight for EWC loss $\lambda_{3}$ to $2\times 10^{4}$. In contrast, if there is no ground truth for training (such as MRI/PET and infrared/visible image fusion tasks in our papers), its loss function is formulated as:

(9) $$L=\lambda_{1}L_{\text{MEF-SSIM}}+\lambda_{2}L_{\mathrm{ewc}}\;,$$

where $\lambda_{1}=1$ and $\lambda_{2}$ is set to $2\times 10^{4}$. Particularly, as the first trained task, we set $\lambda_{2}=0$ for infrared/visible image fusion, which means there is no importance degree measurement for it.

### 3.3. Training Data Preparation

It is challenging to get ground-truth all-in-focus images when training for the multi-focus image fusion task. Thus the synthetic data is usually served as the training data. A typical approach is applying the Gaussian filtering with different covariance on a clear image by the defined mask patterns to generate a complementary pair of defocused images. The original clear image is used as the ground truth for comparison with the fused image [6]. However, this method can only provide two defocus images, which is insufficient for more than two images. Therefore, we proposed a pattern-based data synthetic approach to generate multiple defocused images for multi-focus image fusion training.

**Figure 5:**
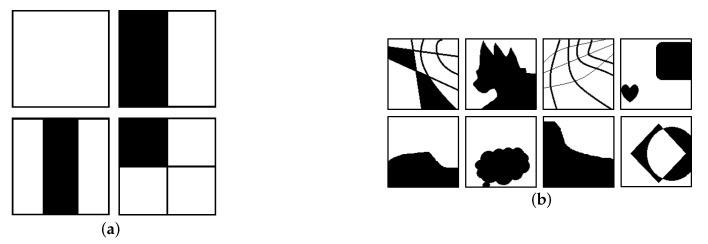
Four basal masks and some examples of shape masks. (**a**) Basal masks, (**b**) Shape masks.

**Figure 6:**
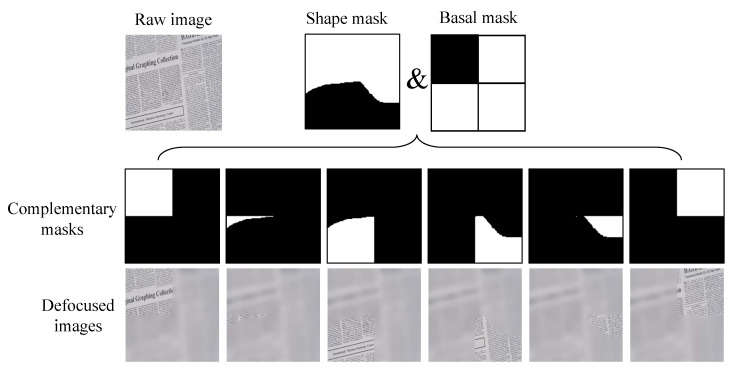
Generation of the defocused images.

Here, we take the dataset DIV2k [42] as the raw images to synthesize the defocused images. The DIV2k dataset contains 1000 high definition high-resolution images with a large diversity of contents. We generate a series of binary masks to decide the out-of-focus area of defocused images. The number of binary masks decides the number of defocused images in each set. To generate such binary complimentary masks, we design two groups of masks in advance. The first group is basal masks, including 4 binary masks as shown in Figure 5a, which separate a 128×128 patch into 1, 2, 3 and 4 parts, respectively. The other group includes 213 binary masks of various shapes with the size of 128×128, called shape masks (Figure 5b). The complementary masks can be defined by one basal mask and a chosen shape mask with AND-operation. Figure 6 gives an example of the binary complementary masks $P_{i}\left(i=1,2,\ldots ,n\right)$. We take the high-resolution image from the DIV2k dataset and crop a 128×128 image patch as the raw clear image, denoted as $I_{clear}$. The Gaussian filtering with a standard deviation between 1 and 2 is applied to get the blurred image $I_{blur}$. Then the *i*-th defocused image $D_{i}$ is generated by the following operation:

(10) $$D_{i}\left(x,y\right)=I_{clear}\left(x,y\right)P_{i}\left(x,y\right)+I_{blur}\left(x,y\right)\left(1-P_{i}\left(x,y\right)\right)\;,$$

where *M* denotes the total number of defocused images, *x* and *y* are pixel coordinates. In this way, we prepare 3000 multi-focus image sets, each containing 2 to 8 defocused images and a ground truth all-in-focus image with the size of 128×128 for training.

### 3.4. Implementation Details

We collect 220 pairs of MRI/PET images on Havard Medical School (https://www.med.harvard.edu/aanlib/home.html (accessed on 13 February 2022)) from different medical cases of illness and divide them into a training set (200 pairs) and test set (20 pairs). As for infrared/visible image fusion, we adopt RoadScene (https://github.com/jiayi-ma/RoadScene (accessed on 13 February 2022)) for training. This dataset contains 221 aligned infrared/visible image pairs containing rich scenes. In addition, we use datasets from [43] for multi-exposure image fusion training, which provides multi-exposure sequences of 589 scenes with 3 to 18 exposure levels as well as corresponding high-quality reference images. The reference HDR image is the best fusion image manually selected from the fusion results of 13 recently developed MEF and HDR algorithms that are used as ground truth during training. The training data for multi-focus image fusion is generated according to Section 3.3. All source images are cropped to image patches with a size of 128×128. The filters size of the network are set to 3×3.

Images in different fusion tasks have different color channels. For instance, the multi-exposure images can be all RGB images or gray-scale images. In contrast, the infrared/visible images usually contain an RGB visible image and a gray-scale infrared image. As we all know, the image structural details are present in the luminance channel, and the brightness variation is prominent in the luminance channel than in chrominance channels. It is a popular method to handle image fusion in YCbCr color space [7]. To unify the input color channel for all fusion tasks, we convert all RGB images into the YCbCr format and only feed the Y channel (brightness) into the CNN since the Y channel carries almost all detailed information of images. Then the chrominance information Cb (or Cr) of the output fused image is generated by the weighted sum of input chrominance channel values [7,44]:

(11) $$C_{f}=\left\{\begin{array}{ccc} \frac{\sum_{i}^{N}C_{i}\left(\left|C_{i}-\tau \right|\right)}{\sum_{i}^{N}\left(\left|C_{i}-\tau \right|\right)}, & \; & \sum_{i}^{N}\left(\left|C_{i}-\tau \right|\right)\neq 0\\ \tau , & \; & \mathrm{otherwise} \end{array}\right.\;,$$

where *N* is the number of input images, $C_{i}$ denotes Cb (or Cr) channel of the *i*-th image and $C_{f}$ is the fused channel. τ=128 represents the mid-intensity value for the 8-bit image, and $C_{f}$ is defined as 128 when all $C_{i}=128$.

Our IFANI is implemented by PyTorch on the CPU of Intel Core i7-8086k 195 of 32 GB RAM and the GPU of NVIDIA GTX 1080Ti. We use the Adam method with $\beta_{1}=0.9$ and $\beta_{2}=0.99$ for optimization. These four tasks orderly train for 300, 200, 270 and 3 epochs with batch size of 1. The learning rate starts at $1\times 10^{-4}$ and reduces to half after every $5\times 10^{4}$ iterations.

## 4. Experiments

In this section, we conduct objective and subjective experiments on four fusion tasks to demonstrate the effectiveness of our method. Furthermore, we perform a set of ablation studies to explore the effect of different elements of the proposed IFANI.

### 4.1. Multi-Modality Medical Image Fusion

Test data of MRI/PET images come from Harvard Medical School. We compare our method with four state-of-the-art methods, including a traditional fusion method [45] and three DL-based fusion methods, PMGI [46], FusionDN [33] and U2Fusion [8]. Table 2 presents the quantitative comparison results between our method and the other state-of-the-art methods based on the sum of the correlations of differences (SCD) [47], the average structural similarity (SSIM) [41], MEF-SSIM [30], the visual information fidelity (VIF) [48], and the mutual information (FMI) [49]. Note that all metrics are designed based on gray images, so we compute them in the luminance channel, and the same goes for the other fusion tasks that follow.

**Figure 7:**
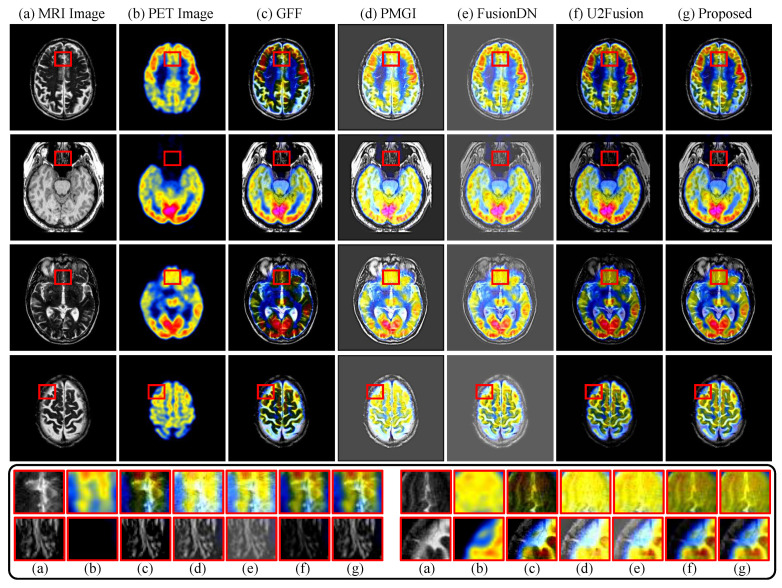
Fusion results for PET/MRI images on Havard dataset of our proposed method and four image fusion methods: GFF [45], PMGI [46], FusionDN [33] and U2Fusion [8]. The zoom-in patches of corresponding images (**a**–**g**) are shown in the black box.

In Table 2, our IFANI achieves the highest SCD, MEF-SSIM, and VIF results among all the methods. Our method also gets the second-best performance in terms of SSIM and FMI. Figure 7 visualizes the fused results using different methods for MRI/PET pairs. It shows that PMGI and FusionDN fused the black pixels into gray pixels, which means they have limited fusion ability for such a special situation. Trained with MRI/PET images, this problem also appears in U2Fusion in less severe. In contrast, our method reconstructed the fused image with clearer tissue structures and can successfully handle the black areas. Moreover, our method maintains more details from the PET images than GFF and more structures from the MRI images than U2Fusion.

The average running times of GFF, PMGI, FusionDN, U2Fusion and the proposed IFANI are around 0.023, 0.057, 4.506, 1.052 and 0.068 s respectively.

### 4.2. Infrared/Visible Image Fusion

For the task of visible and infrared image fusion, we take image datasets TNO (https://figshare.com/articles/dataset/TNO_Image_Fusion_Dataset/1008029 (accessed on 13 February 2022)) (20 pairs) and RoadScene (20 pairs) for testing. The comparison methods include a traditional fusion method GFF [45], a specialized infrared/visible image fusion method Densefuse [5] and three general DL-based methods IFCNN [32], PMGI [46] and U2Fusion [8].

**Figure 8:**
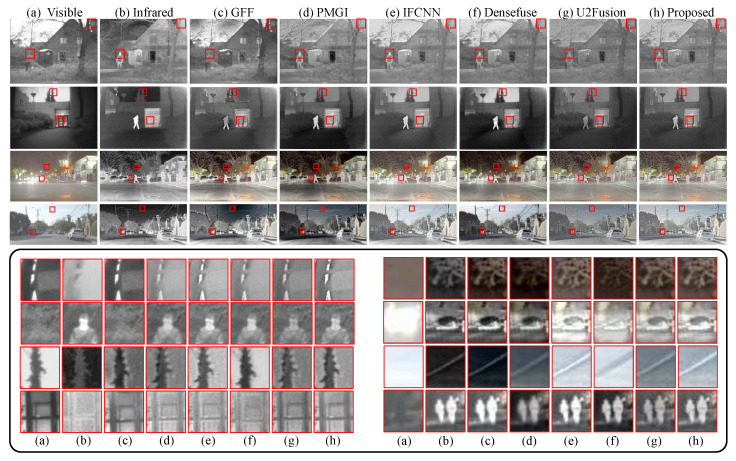
Infrared and visible image fusion results of our proposed method and other five methods (GFF [45], PMGI [46], IFCNN [32] and Densefuse [5]). Images in the top two rows come from the TNO dataset, and images in the bottom two lines come from the RoadScene dataset. The zoom-in patches of corresponding images (**a**–**h**) are shown in the black box.

As can be seen in Table 3, we measure the fusion results on 4 metrics: edge information preservation ($Q_{M}$) [50], weighted SSIM ($Q_{Y}$) [51], pixel intensity correlation ($Q^{AB/F}$) [52], and MEF-SSIM. Our method outperforms other methods on the TNO dataset, ranking first for all metrics. Besides, it achieves the highest scores on RoadScene in terms of $Q^{AB/F}$ and MEF-SSIM and the second-ranking for $Q_{M}$ and $Q_{Y}$. GFF [45], as a traditional image fusion method, fuses images with high quality, which gets two first-rankings and 4 second-rankings on two datasets. In Figure 8, we visualize the fused images using different methods. Usually, the infrared images can help recognize humans, especially when the luminance condition of the scene is poor. In contrast, the visible image contains more informative environment details than infrared images. From Figure 8 we can see our method can fuse clear pedestrians and informative environment details for 4 different scenes. On the contrary, GFF captures more information from the infrared images, losing environment textures in the RoadScene dataset. The same problems happen to PMGI. The specialized infrared/visible image fusion method Densefuse shows outstanding reconstruction ability for the daylight sky, but it loses the environment information in the night scene (3rd line). In comparison, our method has good fusion performance for images of day scenes and night scenes.

Moreover, the average running times of GFF, IFCNN, Densefuse, PMGI, U2Fusion and the proposed IFANI are around 0.093, 0.046, 0.725, 0.301, 2.034, and 0.129 s, respectively.

### 4.3. Multi-Exposure Image Fusion

In the multi-exposure image fusion task, we compare our method with four image fusion methods, including the specialized multi-exposure image fusion method FMMEF [53] and three general image fusion methods: GFF [45], IFCNN [32] and U2Fusion [8]. The comparison is implemented on 17 multi-exposure image sets in [30]. All sets contain at least three input images captured from underexposed to overexposed cases. We measure the quality of the fused images by the information-based metric called MEF-VIF [54] as well as MEF-SSIM [30]. Here, the MEF-VIF and MEF-SSIM metrics are widely used to measure the perceptual quality of fused images of the multi-exposure image fusion task and apply for multiple source images.

**Figure 9:**
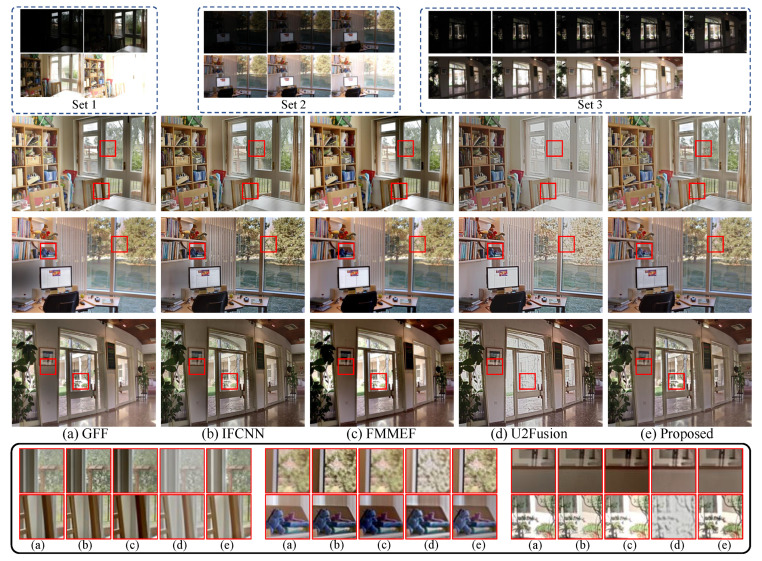
Multi-exposure image fusion results of four image fusion methods (GFF [45], IFCNN [32], FMMEF [53], U2Fusion [8]) and our proposed method. The zoom-in patches of corresponding images (**a**–**e**) are shown in the black box.

As shown in Table 4, our method achieves the highest average scores on MEF-SSIM and VIF-SSIM and outperforms other fusion methods on most sets. GFF and FMMEF achieve the second good average scores on VIF-SSIM. U2Fusion gets lower average scores than other fusion methods on both MEF-SSIM and VIF-SSIM. We believe that the fusion strategy is one of the reasons for the poor fusion results, which will be further explored in the oblation study. The subjective comparisons in Figure 9 show consistent results with the objective results. As can be seen, we fuse 3 multi-exposure image sets that have 4, 6, and 9 low dynamic range images in each set (first row). U2Fusion loses several details of the outdoor scenes for 3 sets, while our method can preserve them successfully. GFF, IFCNN, and IFMEF fuse images from set 1 with non-ideal shadow. In contrast, the fused images from our method have vivid color.

**Figure 10:**
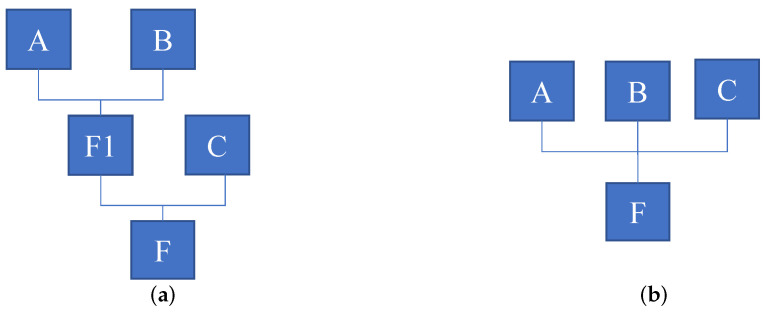
Two fusion strategies for more than two input images. Our method takes one-shot fusion strategy to fused the image. (**a**) Recursive fusion, (**b**) Our fusion.

As introduced in Section 3, our method is permutation-invariant for multiple inputs and can fuse a set of images at once, as illustrated in Figure 10b. It treats all images without distinction and has a global measuring ability. However, other DL-based methods such as IFCNN and U2Fusion are designed for two inputs; they adopt a recursive fusion strategy to get the fused image. More specifically, they initially fuse two of these source images, and then the intermediate result is fused with another source image (Figure 10a). That means the third image is only compared with the intermediate fused image rather than the source images, resulting in the lack of global measurement for the network for all inputs. We will further explore the impact of different fusion strategies in Section 4.5.2.

The average running times of GFF, IFCNN, U2Fusion, FMMEF and the proposed method are about 0.202, 0.323, 10.661, 0.086 and 0.292 s, respectively. Obviously, U2Fusion and IFCNN using the recursive fusion strategy are time-consuming.

### 4.4. Multi-Focus Image Fusion

We evaluate our proposed method for the multi-focus image fusion task on two image datasets. The first one is the Lytro (https://mansournejati.ece.iut.ac.ir/content/lytro-multi-focus-dataset (accessed on 13 February 2022)) dataset that contains 20 pairs of color multi-focus images and four series of color multi-focus images with 3 sources. The second one is the grayscale multi-focus dataset used in [55], which contains 8 pairs of grayscale images.

Our proposed method is compared with five image fusion methods, including the specialized multi-focus image fusion method SESF [28] and four general fusion methods GFF [45], IFCNN [32], PMGI [46] and U2Fusion [8]. We take assessments based on image entropy (EN), mutual information (MI), structure similarity (MEF-SSIM) [30] and pixel intensity correlation $Q^{AB/F}$. As shown in Table 5, we compare the fusion results of the 20 pairs on the Lytro dataset and find our proposed method outperforms other fusion methods on most assessment metrics except for MEF-SSIM, while GFF and SESF achieve the highest scores on MEF-SSIM. As for the grayscale dataset, our method ranks first for all metrics.

**Figure 11:**
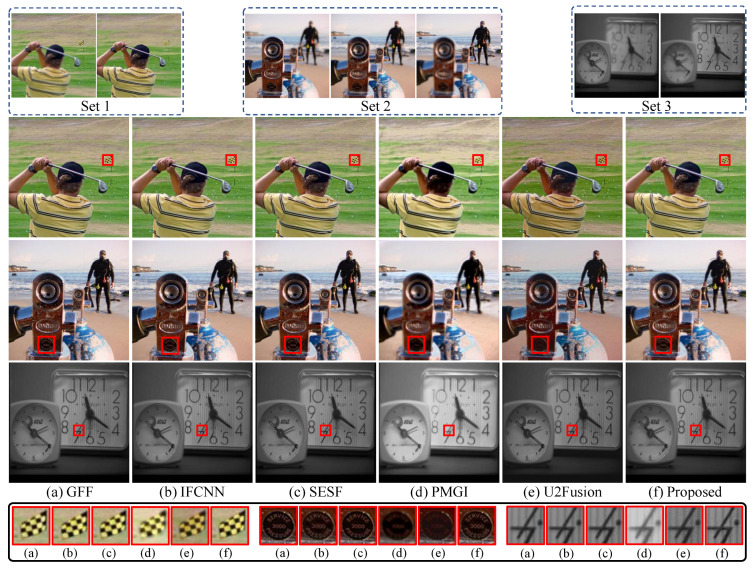
Multi-focus image fusion results of the proposed IFANI and other five image fusion methods (GFF [45], IFCNN [32], SESF [28], PMGI [46], and U2Fusion [8]) on the Lytro and the grayscale datasets. The zoom-in patches of corresponding images (**a**–**f**) are shown in the black box.

We subjectively compare these fusion methods in Figure 11. We also compare the multi-focus sets with 3 images in Lytro (set 2). In the third row of Figure 11, PMGI and U2Fusion fuse the multi-focus images to a blurred image, where the words are unclear on the plate. However, our method fuses the image with clear background and foreground, and the words on the plate are clear. It confirms that our method can fully use all defocused images to fuse a clear all-in-focus image.

The average running times of GFF, IFCNN, SESF, PMGI, U2Fusion and the proposed method are 0.106, 0.064, 0.347, 0.159, 3.087 and 0.288 s, respectively.

### 4.5. Ablation Study

In this section, we explore the effect of different elements in the pipeline of the proposed IFANI by a sequence of controlled experiments.

#### 4.5.1. Symmetry Function

**Figure 12:**
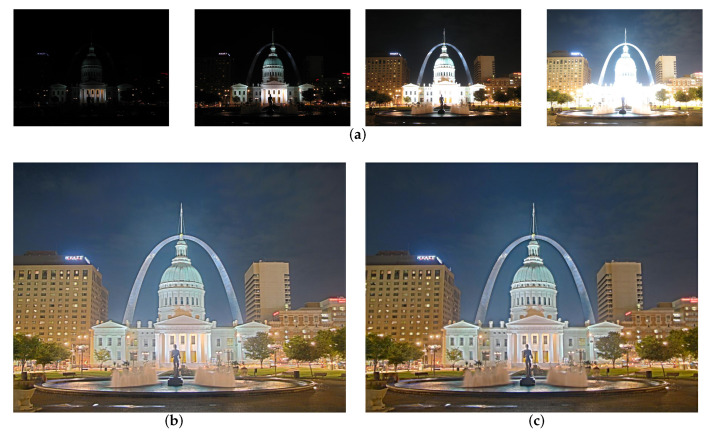
Multi-exposure image fusion results for networks with sum-pooling and max-pooling. (**a**) Multi-exposure image set, (**b**) Sum-pooling fused image, (**c**) Max-pooling fused image.

There are mainly two symmetry functions for achieving the permutation-invariance of the network, including sum-pooling and max-pooling. The sum pooling adds all features of every input image together, while the max-pooling selects the most significant features among them. We take the comparisons of different symmetry functions on the multi-exposure image fusion task. Note that all network settings and training procedures are the same for the two networks except for the pooling strategies. As shown in Table 6, images fused by max-pooling have higher average MEF-SSIM and MEF-VIF than images fused by the sum-pooling, which is consistent with the subjective results (Figure 12). The fused image by the max-pooling network (Figure 12c) has uniform brightness and clear contrast. In addition, it preserves more details than the image fused by the sum-pooling network (Figure 12b). Therefore, the proposed IFANI chooses the max-pooling to enable permutation-invariance.

#### 4.5.2. Input Order

To investigate the influence of different orders of input images on the proposed IFANI, we compare our method with U2Fusion [8] on the multi-exposure image fusion task. As mentioned in Section 4.3, U2Fusion is designed for two inputs. Therefore, it adopts the recursive fusion strategy to fuse more than two source images (Figure 10a). In contrast, our method can fuse all source images in one shot(Figure 10b).

**Figure 13:**
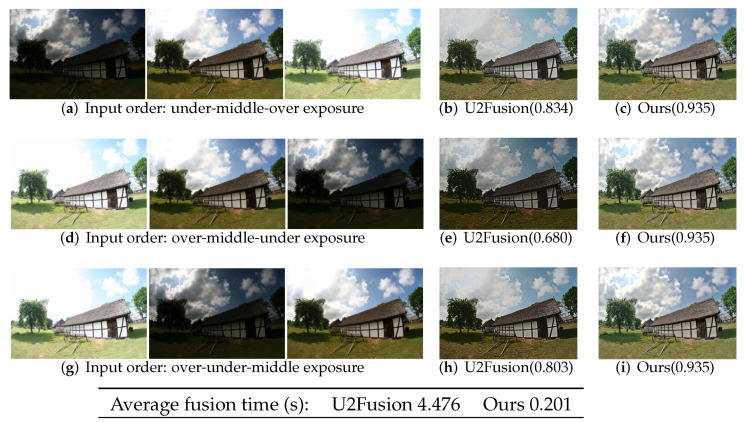
Multi-exposure image fusion results for different sequence orders (**a**,**d**,**g**) by U2Fusion (**b**,**e**,**h**) and our method (**c**,**f**,**i**). The values in parentheses are MEF-SSIM [30] assessments of fused images. The average running time of U2Fusion for each input set is 4.476 s, while ours if 0.201 s.

In Figure 13, the multi-exposure set contains 3 images with under, middle, and over exposures. For image order in Figure 13a, U2Fusion first fuses the under-exposure and middle-exposure images. Then the intermediate output is combined with the under-exposure image to obtain the final fused image (Figure 13c). This recursive fusion also applies for image order of Figure 13b,g and for U2Fusion to get fused images Figure 13e,h. Our method handles these three orders in the same way (Figure 10b) and obtains the corresponding fused images in Figure 13d,f,i. As can be seen, the fused images of U2Fusion for three input orders have big differences and have lower quality than our fused images according to MEF-SSIM [30] assessments. Lacking the global vision of all source images, U2Fusion can not extract the most important details among all images. By contrast, our method is permutation-invariant that can process all images in one shot without remembering their orders. It obtains the same and also high-quality fused images for different input orders. Moreover, our one-shot fusion method is more time-efficient (0.201 s/set) than the recursive fusion strategy (4.476 s/set).

#### 4.5.3. Continual Learning

Our IFANI uses EWC-based continual learning to train multiple tasks. We explore the effectiveness of continual learning by training with multi-exposure image fusion task and multi-focus image fusion task on 3 model settings, as shown in Table 7. Model 1 is only trained with multi-exposure task. Model 2 is first trained with multi-exposure task and then trained with multi-focus task without EWC-based continual learning. In contrast, model 3 is continually trained with multi-focus image fusion task based on EWC.

**Figure 14:**
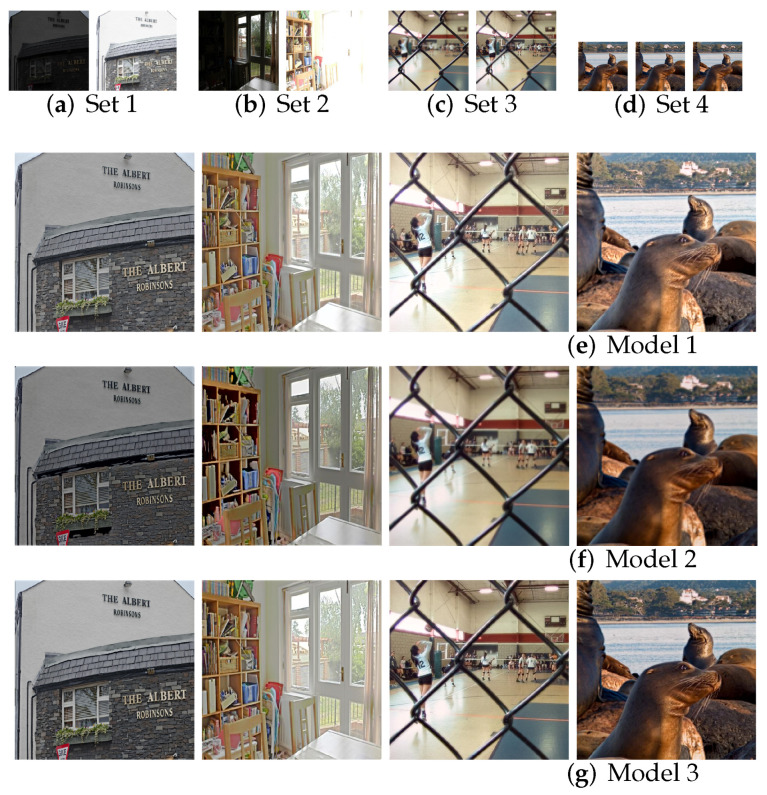
Comparison results of multi-exposure and multi-focus image fusion with and without the continual learning based on EWC. Sets 1 and 2 are for the multi-exposure task; sets 3 and 4 are for the multi-focus task.

We compare the fusion results of two fusion tasks by MEF-SSIM. Model 1 obtains the highest MEF-SSIM (0.910) for the multi-exposure image fusion task but the lowest performance (0.932) for the multi-focus image fusion task. That’s because it has no knowledge about multi-focus images. In contrast, model 2 gets higher MEF-SSIM results than model 1 in multi-focus image fusion. However, without EWC, model 2 losses the knowledge of the previous task and gets the worst performance for the multi-exposure image fusion task after training with the multi-focus image fusion task. As for model 3, it achieves the best performance in the multi-focus image fusion. Simultaneously, its performance for the multi-exposure image fusion only reduces 0.002 compared with model 1, which means model 3 remembers most information from the previous task by the EWC-based continual learning. In addition, model 3 outperforms model 2 on the multi-focus image fusion task, which means the knowledge of the multi-exposure task also contributes to learning the multi-focus image fusion. The subjective comparison is in Figure 14. With EWC, model 3 gets the HDR images with more consistent and uniform brightness across the entire image than that in model 1. Note that model 2 loses the fusion ability for the multi-exposure image fusion after retraining the multi-focus image fusion task without the EWC; thus, its fused HDR image suffers severe hole effects. These experiments show that the EWC-based continual learning not only maintains knowledge from the previous task but also promotes the training of the current task.

## 5. Conclusions

This paper addresses the general image fusion problems with an arbitrary number of inputs by proposing a novel and permutation-invariant neural network named IFANI. Different from other deep learning-based image fusion methods that can only apply to two inputs, our method can fuse multiple inputs in one shot. Specifically, max-pooling is repeatedly used in different stages of the network to aggregate salient information among all input features. Since the permutation-invariance of max-pooling, the network is permutation-invariant so that it can fuse several images regardless of their input orders and numbers, providing convenience during the inference of networks. The continual learning based on Elastic Weight Consolidation is adopted to remember the knowledge of the previous task during the training of the current task. By this means, we can handle four different fusion tasks with one unified model. Both image quality loss and EWC loss are used for training. The subjective and objective experiments conducted on several public datasets demonstrate the high performance of our IFANI on four fusion tasks. The proposed IFANI mainly works with aligned images or static scenes (e.g., multi-exposure image fusion), and extending it to dynamic scenes is valuable. However, the lack of ground truth images and perceptual image quality metrics of dynamic scenes for networks training is another challenge and worth research in the future.

## Figures and Tables

**Table 1:** List of network architecture information and their trainable parameters, where *N* is the number of input images.

Fusion Block	Network Architecture	Layer Type	Filter Size	Input Channel	Output Channel	Number of Parameters
1	Conv.	ConvLayer+ReLU+BN	3×3	1	32	352
	Pool	MaxPool	1×1	32N	32	-
2	Conv.	ConvLayer+ReLU+BN	3×3	64	32	18,496
	Pool	MaxPool	1×1	32N	32	-
3	Conv.	ConvLayer+ReLU+BN	3×3	64	32	18,496
	Pool	MaxPool	1×1	32N	32	-
4	Conv.	ConvLayer+ReLU+BN	3×3	64	32	18,496
	Pool	MaxPool	1×1	32N	32	-
Bottleneck	Conv.	ConvLayer+ReLU+BN	3×3	64	32	18,496
	Conv.	ConvLayer+ReLU+BN	3×3	32	32	9280
	Conv.	ConvLayer	3×3	32	32	9248
5	Conv.	ConvLayer+ReLU+BN	3×3	32	32	9280
	Pool	MaxPool	1×1	32N	32	-
6	Conv.	ConvLayer+ReLU+BN	3×3	96	32	27,712
	Pool	MaxPool	1×1	32N	32	-
7	Conv.	ConvLayer+ReLU+BN	3×3	96	32	27,712
	Pool	MaxPool	1×1	32N	32	-
8	Conv.	ConvLayer+ReLU+BN	3×3	96	32	27,712
	Pool	MaxPool	1×1	32N	32	-
Tail	Conv.	ConvLayer+ReLU+BN	3×3	96	32	27,712
	Pool	MaxPool	1×1	32N	32	-
	Conv.	ConvLayer+ReLU+BN	3×3	32	1	321

**Table 2:** Comparison results of MRI/PET images fusion on Harvard dataset, with the best result in bold and the second-best result underlined.

Method	SCD	SSIM	MEF-SSIM	VIF	FMI
GFF [45]	0.850	**0.764**	0.873	0.414	**0.559**
PMGI [46]	1.096	0.248	0.874	0.387	0.376
U2Fusion [8]	0.283	0.226	0.837	0.232	0.336
FusionDN [33]	0.520	0.255	0.864	0.331	0.353
Proposed	**1.117**	0.746	**0.938**	**0.467**	0.405

**Table 3:** Comparison results of infrared/visible image fusion on TNO and RoadScene datasets, with the best results in bold and the second-best results underlined.

Dataset	Method	$Q_{M}$	$Q_{Y}$	$Q^{\mathrm{AB}/F}$	MEF-SSIM
TNO	GFF [45]	2.921	0.739	0.440	0.826
	IFCNN [32]	2.915	0.709	0.418	0.910
	Densefuse [5]	2.898	0.664	0.388	0.862
	PMGI [46]	2.884	0.617	0.331	0.842
	U2Fusion [8]	2.882	0.655	0.356	0.901
	Proposed	**2.928**	**0.836**	**0.539**	**0.929**
RoadScene	GFF [45]	**2.931**	**0.930**	0.594	0.905
	IFCNN [32]	2.906	0.828	0.559	0.906
	Densefuse [5]	2.883	0.756	0.528	0.860
	PMGI [46]	2.871	0.750	0.468	0.903
	U2Fusion [8]	2.872	0.780	0.499	0.886
	Proposed	2.922	0.846	**0.611**	**0.929**

**Table 4:** Comparison results of multi-exposure fusion by MEF-SSIM [30]/MEF-VIF [54], with the best results in bold and the second-best results underlined.

Image Set	GFF [45]	IFCNN [32]	U2Fusion [8]	FMMEF [53]	Proposed
Balloons	0.896/0.891	0.873/0.868	0.803/0.450	0.876/0.886	0.927/0.918
Belgium house	0.921/0.906	0.877/0.888	0.837/0.667	0.830/0.878	0.917/0.926
Lamp1	0.894/0.880	0.876/0.843	0.802/0.611	0.856/0.883	0.894/0.887
Candle	0.844/0.866	0.898/0.872	0.236/0.830	0.914/0.871	0.897/0.836
Cave	0.929/0.937	0.889/0.895	0.246/0.793	0.852/0.933	0.939/0.976
Chinese garden	0.945/0.953	0.882/0.834	0.530/0.835	0.868/0.948	0.938/0.978
Farmhouse	0.948/0.955	0.875/0.906	0.746/0.699	0.858/0.952	0.913/0.983
House	0.869/0.900	0.869/0.853	0.811/0.703	0.861/0.898	0.895/0.912
Kluki	0.894/0.927	0.845/0.870	0.834/0.758	0.886/0.915	0.936/0.934
Lamp2	0.849/0.799	0.793/0.844	0.320/0.729	0.771/0.820	0.850/0.832
Landscape	0.915/0.934	0.809/0.810	0.677/0.885	0.925/0.944	0.956/0.989
Lighthouse	0.877/0.821	0.870/0.763	0.767/0.845	0.918/0.863	0.939/0.851
Madison capitol	0.908/0.914	0.877/0.886	0.780/0.626	0.885/0.928	0.939/0.952
Memorial	0.889/0.886	0.888/0.894	0.130/0.838	0.853/0.858	0.939/0.928
Office	0.906/0.953	0.832/0.870	0.825/0.772	0.899/0.957	0.935/0.972
Tower	0.935/0.947	0.851/0.868	0.568/0.763	0.904/0.943	0.941/0.986
Venice	0.895/0.897	0.835/0.853	0.587/0.740	0.842/0.899	0.921/0.922
Average	0.901/0.904	0.861/0.860	0.618/0.738	0.870/0.904	**0.922/0.928**

**Table 5:** Comparison results of multi-focus image fusion on the Lytro and the grayscale datasets, with the best results in bold and the second-best results underlined.

Dataset	Method	EN	MI	MEF-SSIM	$Q^{\mathrm{AB}/F}$
Lytro	GFF [45]	7.535	15.069	**0.994**	0.564
	IFCNN [32]	7.534	15.068	0.993	0.684
	SESF [28]	7.534	15.067	**0.994**	0.536
	PMGI [46]	7.517	15.034	0.958	0.539
	U2Fusion [8]	7.288	14.575	0.970	0.638
	Proposed	**7.539**	**15.077**	0.991	**0.716**
grayscale	GFF [45]	7.255	14.510	**0.989**	0.500
	IFCNN [32]	7.245	14.489	0.986	0.656
	SESF [28]	7.243	14.486	0.988	0.432
	PMGI [46]	7.207	14.414	0.959	0.548
	U2Fusion [8]	7.090	14.180	0.974	0.618
	Proposed	**7.260**	**14.520**	**0.989**	**0.682**

**Table 6:** Average results of MEF-SSIM [30] and MEF-VIF [54] for networks with two symmetry functions on multi-exposure image fusion task.

Symmetry Function	MEF-SSIM	MEF-VIF
Sum-pooling	0.902	0.901
Max-pooling	**0.922**	**0.928**

**Table 7:** Comparison results of multi-exposure and multi-focus image fusions with and without the continual learning based on EWC.

Model	Task Training		Continual Learning		Test Results (MEF-SSIM)	
	Multi-Exposure	Multi-Focus	w/o EWC	with EWC	Multi-Exposure	Multi-Focus
1	✓	×	×	×	0.910	0.932
2	✓	✓	✓	×	0.754	0.979
3	✓	✓	×	✓	0.908	0.988

## Data Availability

No new data were created or analyzed in this study. Data sharing is not applicable to this article.
